# Multiplex array analysis of serum cytokines offers minimal predictive value for cognitive function in the subacute phase after stroke

**DOI:** 10.3389/fneur.2022.886018

**Published:** 2022-10-18

**Authors:** Yuling Zhang, Haixin Song, Jun Wang, Xiao Xi, Philip Cefalo, Lisa J. Wood, Xun Luo, Qing Mei Wang

**Affiliations:** ^1^Stroke Biological Recovery Laboratory, Spaulding Rehabilitation Hospital, The Teaching Affiliate of Harvard Medical School, Charlestown, MA, United States; ^2^School of Health Science and Engineering, University of Shanghai for Science and Technology, Shanghai, China; ^3^Rehabilitation Department, Sir Run Run Show Hospital, Hangzhou, China; ^4^School of Medicine, Shenzhen University, Shenzhen, China; ^5^Department of Rehabilitation Medicine, Xijing Hospital, The Fourth Military Medical University, Xi'an, China; ^6^Department of Physical Medicine and Rehabilitation, Spaulding Rehabilitation Hospital, The Teaching Affiliate of Harvard Medical School, Charlestown, MA, United States; ^7^William F. Connell School of Nursing at Boston College, Boston, MA, United States; ^8^Kerry Rehabilitation Medicine Research Institute, Shenzhen, China

**Keywords:** post-stroke, cognitive function, inflammation, cytokines, multiplex array analysis

## Abstract

**Objective:**

The effects of inflammation on post-stroke cognitive function are still unclear. This study investigated the correlation between the Th17-related cytokines in peripheral blood and post-stroke cognitive function after ischemic stroke in the subacute phase.

**Design:**

A retrospective cohort study.

**Setting:**

Academic acute inpatient rehabilitation facility.

**Participants:**

One hundred and fourteen patients with first ischemic stroke were categorized as the poor cognitive recovery group (*n* = 58) or good cognitive recovery group (*n* = 56) based on their cognitive MRFS efficiency.

**Interventions:**

All subjects received routine physical, occupational, and speech-language pathology therapy.

**Main outcome measures:**

Serum cytokines/chemokine (IL-1 β, IL-2, IL-4, IL-5, IL-6, IL-9, IL-10, IL-12p70, IL-13, IL-15, IL-17A, IL-17E, IL-17F, IL-21, IL-22, IL-23, IL-27, IL-28A, IL-31, IL-33, GM-CSF, IFN-γ, MIP-3 α, TNF-α, and TNF-β) levels were measured in duplicate using Human Th17 magnetic bead panel and multiplex array analysis (Luminex-200 system). The primary functional outcome was a gain in functional independence measure (FIM) cognitive subscore at discharge. The secondary outcome measures were FIM total score at discharge, length of stay in the hospital, and discharge destination. Cognitive Montebello Rehabilitation Factor Score (MRFS) and cognitive MRFS efficiency were calculated. Demographic and clinical characteristics were obtained from the medical record.

**Results:**

The good cognitive recovery group had an interesting trend of higher IL-13 than the poor cognitive recovery group (good cognitive recovery group 257.82 ± 268.76 vs. poor cognitive recovery group 191.67 ± 201.82, *p* = 0.049, unit: pg/ml). However, Pearson's correlation analysis showed no significant correlation between cytokine levels and gain of cognition, cognitive MRFS, or cognitive MRFS efficiency. Receiver operating characteristic (ROC) analysis of cytokines also suggested a low accuracy of prediction as a predictor for post-stroke cognitive recovery improvement.

**Conclusion:**

Our preliminary findings suggested that the level of serum cytokines had minimal predictive value for the recovery of cognitive function during the subacute inpatient rehabilitation after stroke.

## Introduction

Stroke is the global leading cause of long-term disability (1), resulting not only in physical disability but also in significant cognitive impairment (2, 3). While cognitive impairment and cerebrovascular disease shared some pathophysiologic mechanisms, post-stroke cognitive impairment (PSCI) become one of the common post-stroke complications (4, 5). Post-stroke vascular dementia affects 30% of survivors, and the incidence of new-onset dementia after stroke increases from 7 to 48% in 25 years (6). PSCI can cause direct effects on the quality of life, often resulting in poor functional recovery through poor compliance with treatment guidelines (7). Cognitive function is a prerequisite for functional rehabilitation and played a critical role in patient functional recovery (8). Therefore, for the early prevention of PSCI *via* comprehensive monitoring and interventions, it is urgent to explore precise and reliable biomarkers to effectively predict the risk of PSCI after stroke.

Inflammation is a key component of stroke-related brain injury and has been implicated as an important mechanism underlying cognitive impairment (9). The development of inflammation in stroke is also affected by many factors. Inflammatory cytokines play a crucial role in information transmission, activation and regulation of immune cells, mediating the activation, multiplication, and differentiation of T and B cells and in the inflammatory caspase reaction (10). There is a close relationship between cytokines (such as IL, TNF, and interferons) and the neuroinflammation of ischemic stroke (11–14). However, few data are available regarding their involvement in vascular cognitive impairment (15).

Cytokines are a large group of small signaling proteins which are involved in the whole processing of neuroinflammation (16). To date, the relationship between PSCI and inflammatory cytokines is still controversial. There is a trend of increasing levels of both pro-inflammatory (TNF- α, IL-1β, and IL-6) and anti-inflammatory cytokines (IL-1ra and IL-10) in the plasma of patients with AD (17). Several serum cytokine markers could possibly take part in mediating the inflammatory processes after acute stroke onset. TNF- α, IL-1 β, IL-6, IL-8, IL-12, and IL-10 have been hypothesized to be involved in stoke prognosis (18). This implies that inflammatory cytokines may also serve as a key mediator in the development of PSCI. Few studies have examined the result of the inflammation on cognitive function in a post-stroke setting (18). However, there are limited data linking cytokine levels and PSCI (19).

In many immune-inflammatory diseases, Th17 cell plays a key role in the induction of tissue inflammation and destruction (20). Th17 cells can promote neuroinflammation directly by releasing pro-inflammatory cytokines (IL-17, IL-21, IL22, IL-23, IFN-γ, and GM-CSF) and by inducing neuronal apoptosis through direct cell-cell contact (21). Th17 can penetrate the blood-brain-barrier to become involved in inflammatory response of nervous tissue after ischemic (22). Cytokines from Th17 may be correlated with a gain of cognitive recovery after stroke.

Therefore, this study aimed to explore a potential association between Th17-related cytokines and poststroke cognitive function. We measured the levels of 25 cytokines/chemokines [IL-1 β, IL-2, IL-4, IL-5, IL-6, IL-9, IL-10, IL-12p70, IL-13, IL-15, IL-17A, IL-17E, IL-17F, IL-21, IL-22, IL-23, IL-27, IL-28A, IL-31, IL-33, granulocyte macrophage colony-stimulating factor (GM-CSF), interferon (IFN)-γ, macrophage inflammatory protein (MIP)-3α TNF-α, TNF-β] in serum from patients using Human Th17 magnetic bead panel and multiplex array analysis. In addition, the study also looked into the association between inflammatory markers and post-stroke cognitive outcomes in the subacute phase.

## Subjects and methods

### Subjects

**Figure 1:**
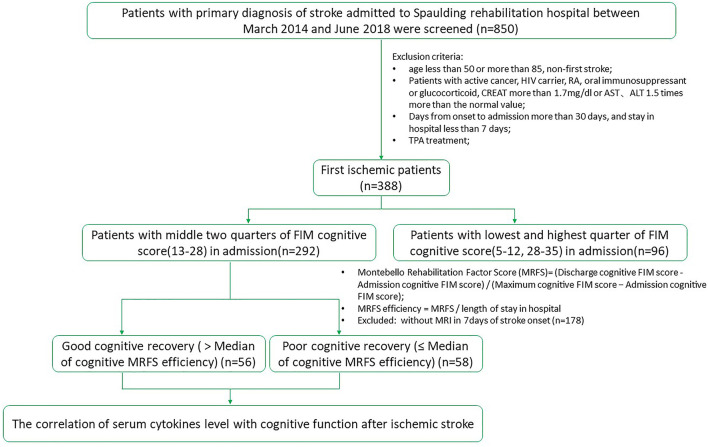
Study flow chart.

This was a retrospective study that was approved by the Institutional Review Board (IRB). Patients with ischemic stroke admitted to the acute inpatient rehabilitation in Spaulding Rehabilitation Hospital from March 2014 to June 2018 were screened. According to the inclusion/exclusion criteria, 114 patients with middle two-quarters of FIM cognitive score (13–28) in admission were recruited. One hundred and fourteen patients with middle two-quarters of FIM cognitive score (13–28) in admission met the inclusion/exclusion criteria and were included in the study. They were divided into two groups, poor and good cognitive recovery groups (Figure 1). The median for cognitive MRFS efficiency was used as the cutoff.

The MRFS efficiency was described in the section “Clinical functional outcome assessment.”

The inclusion criteria were as follows:

1. Age 50–85 years;
2. Clinically and radiographically confirmed first ischemic cerebrovascular accident (CVA), MRI scanned within 7 days of stroke onset;
3. No history of neuropsychiatric diseases, cerebral vascular abnormalities, epilepsy, or trauma;
4. Peripheral blood serum sample collected and stored upon admission.
5. Patients in the subacute phase of ischemic stroke (post-stroke 72 h−6 weeks) (23).

The exclusion criteria were as follows:

1. Age < 50 or >85, non-first stroke;
2. Patients with active cancer, HIV carrier, RA, oral immunosuppressant, or glucocorticoid, CREAT more than 1.7 mg/dl or AST, ALT 1.5 times more than the normal value;
3. Stay in hospital for < 7 days;
4. TPA treatment;
5. Without MRI in 7 days of stroke onset.

### Demographic and clinical data

The demographic and clinical data were collected from the medical records, including age, gender, ethnicity, body mass index (BMI), marital status, AST, ALT, blood urea nitrogen (BUN) and creatinine, stroke site, length of stay in the hospital, discharge destination, admission and discharge functional independence measure (FIM), and stroke risk factors (hypertension, diabetes mellitus, hyperthyroidism, coronary artery disease, atrial fibrillation, smoking).

### Clinical functional outcome assessment

Functional outcomes were evaluated with the FIM. The reliability and validity of the FIM have been well established for patients with stroke. FIM assessment on admission and discharge was a routine clinical assessment for function during the inpatient rehabilitation at this hospital. FIM score is composed of FIM motor and cognitive subscores. FIM cognitive subscore has two items (communication and social cognition) that are related to cognitive functions such as comprehension, expression, social interaction, problem-solving, and memory. The minimum score is 5 points, and the maximum score is 35 points. FIM cognitive subscore was often utilized to assess the cognitive function of patients with post-stroke in stroke research (2–5).

In the current study, FIM cognitive subscores at admission and discharge were obtained from the medical records and were used as the primary functional outcome. According to the cognitive subscores of the FIM and length of stay in the hospital, the recovery of cognitive function was assessed by the Montebello Rehabilitation Factor Score (MRFS) and MRFS efficiency (24–26). These were calculated according to the following formula.

- MRFS_cognition_= (discharge cognitive FIM score – admission cognitive FIM score)/(maximum cognitive FIM score – admission cognitive FIM score);
- MRFS efficiency = MRFS/length of stay in hospital.

MRFS efficiency is generally used to evaluate the degree of the functional improvement.

The FIM scale includes 13 motor items and five cognitive items. Each item is scored from 1 to 7. Score 1 represents the most serious level with complete dependence on others to finish the item, and score 7 represents full independence. The motor, cognitive, and total FIM scores range from 13 to 91, 5 to 35, and 18 to 126, respectively. All were assessed by a trained therapist and a nurse upon admission and discharge.

All patients received the standard stroke rehabilitation program, including daily 3 h of physical therapy (PT), occupational therapy (OT), and speech therapy (ST), along with 24 h of intensive nursing care and medical treatment in an inpatient hospital environment. All the therapies were performed by trained therapists.

### Multiplex array analysis of serum cytokines

All serum samples were treated in the same way from blood collection to storage. Serum samples were aliquoted to avoid multiple freeze-thawing and stored at −80°C until use. Cytokines levels in serum, including IL-1β, IL-2, IL-4, IL-5, IL-6, IL-9, IL-10, IL-12p70, IL-13, IL-15, IL-17A, IL-17E, IL-17F, IL-21, IL-22, IL-23, IL-27, IL-28A, IL-31, IL-33, GM-CSF, IFN-γ, MIP-3α, TNF-α, and TNF-β were measured in duplicates using a bead based immunofluorescence assay. Each step in processing was according to the manufacturer's instructions (Millipore Inc.). Data were collected and analyzed using the Luminex-200 system version 2.3 (Luminex, Austin, TX, USA). Standards provided by the manufacturer were used to generate a standard curve for each sample. The sample concentrations were calculated from the standard curves *via* a 4- or 5-parameter regression formula. The thresholds for analyte detection are in the Supplementary materials.

### Statistical analysis

Statistical analyses were performed using IBM SPSS Statistics version 23.0 (International Business Machines Corp., New York) and all graphs were made in the GraphPad Prism version 8.0.

Kolmogorov–Smirnov tests and visual inspection of the histogram and Q–Q plots were used to confirm the normal distribution of the continuous variables. Serum cytokines concentrations had a skewed distribution and were log-transformed to achieve a normal distribution. We compared the log-transformed serum cytokines concentrations between clinical groups using Student *t*-tests. To compare the clinical variables between the poor and good cognitive recovery groups, the Student's *t*-test was used for continuous data and chi-square test was used for categorical data. The differences between the two groups were considered significant at *p* < 0.05.

Applying the Bonferroni correction in cytokines multiple comparisons (26), *p*-value (0.05) was divided by the number of tests (25) to get the Bonferroni critical value. *p*-value was smaller than 0.002, which would be considered to be significant between the two groups.

Heatmaps were obtained to study the relationship among 25 cytokines, FIM total scores, FIM cognitive scores and FIM motor scores upon discharge and upon admission, and length of stay in hospital, respectively. R program (3.6.1) was used to conduct this analysis.

Estimating network analysis included estimating a statistical model. Therefore, some parameters from the weighted network could be used to represent a weighted network, and using graph theory could analyze the weighted network (27). R program (3.6.1) was used to conduct this analysis. A Gaussian graphical model was utilized for the estimation of the networks. The estimate Network function was applied to detect ordinal variables, compute polychoric (or, if needed, polyserial and Pearson) correlations, and estimate network structures automatically. The plot function was then applied to display the network (28). In the network structures, the width of the edges represents the strength of connections, while the blue (red) edges stand for positive (negative) relationships.

## Results

### The clinical characteristics of the subjects

As shown in Figure 1, 850 patients were screened, and 114 patients with acute stroke were included in this study with their serum cytokines concentrations measured. Subjects were divided into two groups, poor and good cognitive recovery groups. The median for cognitive MRFS efficiency was used as the cutoff. Baseline characteristics are shown in Table 1. There were significant differences in days of stay in hospital (*p* < 0.01), discharge destination (*p* = 0.002), and stroke sides (*p* = 0.009) between the poor and good cognitive recovery groups. 41.4% of patients in the poor cognitive recovery group were discharged to a skilled nursing facility, whereas 85.7% of patients in the good cognitive recovery group were discharged home. 62.1% of patients in the poor cognitive recovery group had stroke on the right side, whereas 42.9% of patients in the good cognitive recovery group had stroke on the left side.

**Table 1:** The clinical characteristics of patients with ischemic stroke.

**Characteristics**	**Poor cognitive recovery group (*n* = 58) mean ±SD or *n* (%)**	**Good cognitive recovery group (*n* = 56) mean ±SD or n (%)**	***p*-Value**
**Demographics**			
Age (year)	70.14 ± 10.31	68.09 ± 9.15	0.265
Male gender	27 (46.6)	36 (64.3)	0.057
Body mass index (BMI, kg/m^2^)	27.64 ± 5.31	28.64 ± 5.51	0.323
Ethnicity (Hispanic or Latino)	5(8.6)	4 (7.1)	1.000
**Risk factors**			
Hypertension	50 (86.2)	43 (76.8)	0.195
Diabetes	22 (37.9)	18 (32.1)	0.517
Hyperthyroidism	2 (3.4)	2 (3.6)	1.000
Coronary artery disease history (CAD)	8 (13.8)	10 (17.9)	0.552
Atrial fibrillation	16 (27.6)	8 (14.3)	0.082
Smoking	23 (39.7)	21(37.5)	0.585
**Laboratory items**			
AST (units/L)	30.49 ± 18.07	27.31 ± 9.81	0.245
ALT (units/L)	37.07 ± 34.11	32.93 ± 15.77	0.406
BUN (mg/dl)	22.57 ± 11.97	21.05 ± 9.15	0.450
CREAT (mg/dl)	1.01 ± 0.53	0.97 ± 0.36	0.628
**Clinical assessment**			
Days from onset to admission	6.83 ± 4.79	6.43 ± 3.43	0.611
Days of stay in hospital	23.88 ± 10.93	16.27 ± 5.03	0.000*
**Discharge destination**			0.002*
Home	33 (56.9)	48 (85.7)	
Skilled nursing facility	24 (41.4)	7 (12.5)	
Acute hospital	1 (1.7)	1 (1.8)	
**Cognitive FIM scores**			
Admission	19.41 ± 4.26	21.36 ± 4.49	0.019
Discharge	23.60 ± 4.29	29.3 ± 3.81	0.000*
Gain of cognitive FIM (discharge FIM – admission FIM)	4.19 ± 3.60	7.95 ± 3.19	0.000*
MRFS	0.27 ± 0.227	0.61 ± 0.216	0.000*
MRFS efficiency	0.01 ± 0.075	0.04 ± 0.019	0.000*
**Stroke features**			
Stroke sites			0.231
Supratentorial	48 (82.8)	41 (73.2)	
Infratentorial	5 (8.6)	11 (19.6)	
Both	5 (8.6)	4 (7.1)	
Stroke sides			0.009*
Left side	18 (31)	24 (42.9)	
Right side	36(62.1)	20 (35.7)	
Both side	4 (6.9)	12 (21.4)	

Continuous variables are presented as mean ± standard deviation, whereas categorical variables are expressed as counts and percentages.

### Comparison of serum levels of inflammatory cytokines

**Figure 2:**
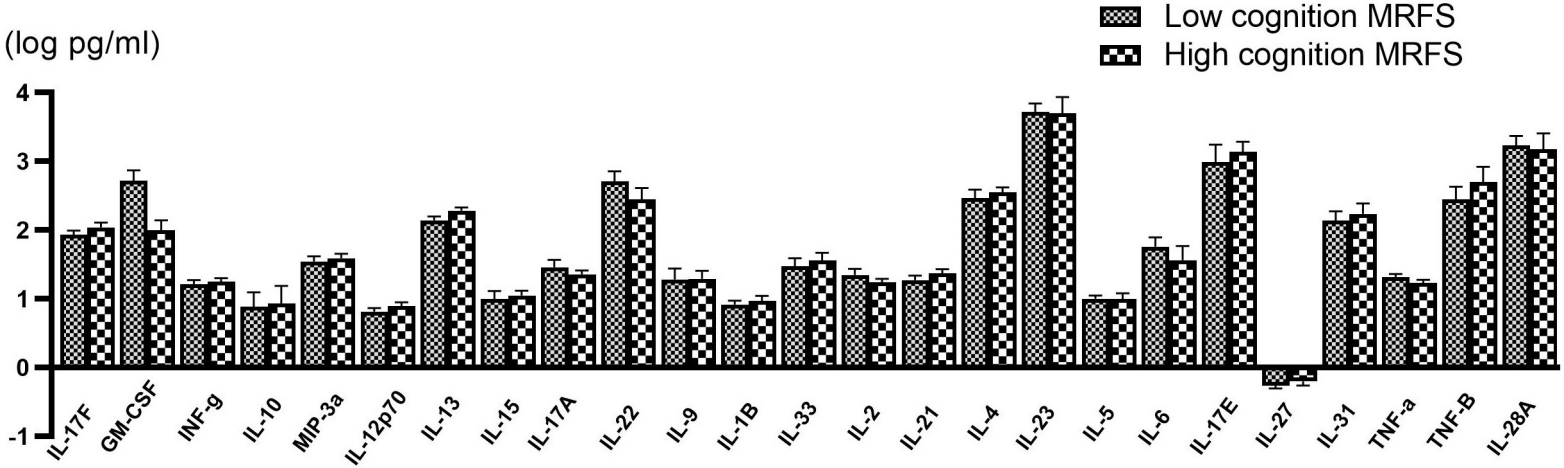
Comparison of various serum cytokine levels between poor (*n* = 58) and good (*n* = 56) cognitive recovery group. Data are presented as mean ± SD.

All serum cytokines detections in the two groups are shown in Table 2. The serum level differences of cytokines are shown in Figure 2.

**Table 2:** The serum cytokines detection in two groups.

**Cytokines**	**Poor cognitive recovery group (*n* = 58) Log mean ±SD (% detectable of total)**	**Good cognitive recovery group (*n* = 56) Log mean ±SD (% detectable of total)**	***p*-Value**
IL-17F	1.9331 ± 0.35699 (60.34%)	2.0418 ± 0.39652 (60.71%)	0.235
GM-CSF	2.7222 ± 0.21351 (3.45%)	1.9989 ± 0.24759 (5.36%)	0.044
IFN-γ	1.2139 ± 0.40664 (79.31%)	1.2487 ± 0.31466 (69.64%)	0.665
IL-10	0.8833 ± 0.64076 (15.52%)	0.9329 ± 0.72268 (14.29%)	0.883
MIP-3α	1.5443 ± 0.54414 (94.83%)	1.5909 ± 0.47406 (100%)	0.631
IL-12p70	0.8155 ± 0.34893 (86.21%)	0.8966 ± 0.37474 (78.57%)	0.280
IL-13	2.1404 ± 0.41121 (93.10%)	2.285 ± 0.34470 (98.21%)	0.049
IL-15	0.999 ± 0.34884 (15.52%)	1.0482 ± 0.26891 (26.79%)	0.701
IL-17A	1.4609 ± 0.35131 (18.97%)	1.3559 ± 0.19599 (19.64%)	0.397
IL-22	2.7141 ± 0.31995 (8.62%)	2.446 ± 0.52571 (17.86%)	0.319
IL-9	1.2835 ± 0.35481 (8.62%)	1.2928 ± 0.026249 (8.93%)	0.964
IL-1 β	0.9194 ± 0.16274 (15.52%)	0.9689 ± 0.24155 (17.86%)	0.611
IL-33	1.472 ± 0.48685 (29.31%)	1.5565 ± 0.51082 (35.71%)	0.612
IL-2	1.3488 ± 0.26232 (15.52%)	1.2411 ± 0.14652 (16.07%)	0.298
IL-21	1.2737 ± 0.39718 (67.24%)	1.3724 ± 0.40313 (76.79%)	0.268
IL-4	2.4698 ± 0.55442 (36.21%)	2.5503 ± 0.38120 (50%)	0.550
IL-23	3.7171 ± 0.41659 (18.97%)	3.7003 ± 0.75100 (17.86%)	0.949
IL-5	1.0021 ± 0.18805 (29.31%)	0.9971 ± 0.25580 (16.07%)	0.955
IL-6	1.7602 ± 0.57675 (31.03%)	1.5625 ± 0.84128 (30.36%)	0.421
IL-17E	2.9932 ± 0.25182 (6.90%)	3.1369 ± 0.39888 (12.5%)	0.536
IL-27	−0.2611 ± 0.31554 (94.83%)	−0.197 ± 0.45100 (100%)	0.388
IL-31	2.1381 ± 0.33095 (10.34%)	2.2339 ± 0.44385 (14.29%)	0.666
TNF-α	1.3138 ± 0.32588 (86.21%)	1.2322 ± 0.33829 (92.86%)	0.218
TNF-β	2.4505 ± 0.51362 (13.79%)	2.699 ± 0.58891 (12.5%)	0.398
IL-28A	3.2374 ± 0.40305 (15.52%)	3.1804 ± 0.60877 (12.5%)	0.825

When the Bonferroni correction is applied, there is no significant difference in serum IL-13 concentration between the two groups. However, IL-13 in the good cognitive recovery group showed a trend of higher concentration levels (257.82 ± 268.76 pg/ml vs. 191.67 ± 201.82 pg/ml, *p* = 0.049 > 0.002). Pearson's correlation analysis showed no significant correlation between IL-13 and gain of cognition (*r* = −0.037, *p* > 0.05), cognitive MRFS (*r* = −0.036, *p* > 0.05), or cognitive MRFS efficiency (*r* = 0.018, *p* > 0.05). GM-CSF cytokine level also showed a slight difference between the two groups (*p* = 0.044), while Pearson's correlation analysis showed no significant correlation between GM-CSF and gain of cognition (*r* = 0.033, *p* > 0.05), cognitive MRFS (*r* = −0.015, *p* > 0.05), or cognitive MRFS efficiency (*r* = −0.035, *p* > 0.05). However, the detectable rate of GM-CSF was very low (3.45% in the poor cognitive recovery group, and 5.36% in the good cognitive recovery group). For the other serum cytokines, there is no significant difference between the two groups.

**Figure 3:**
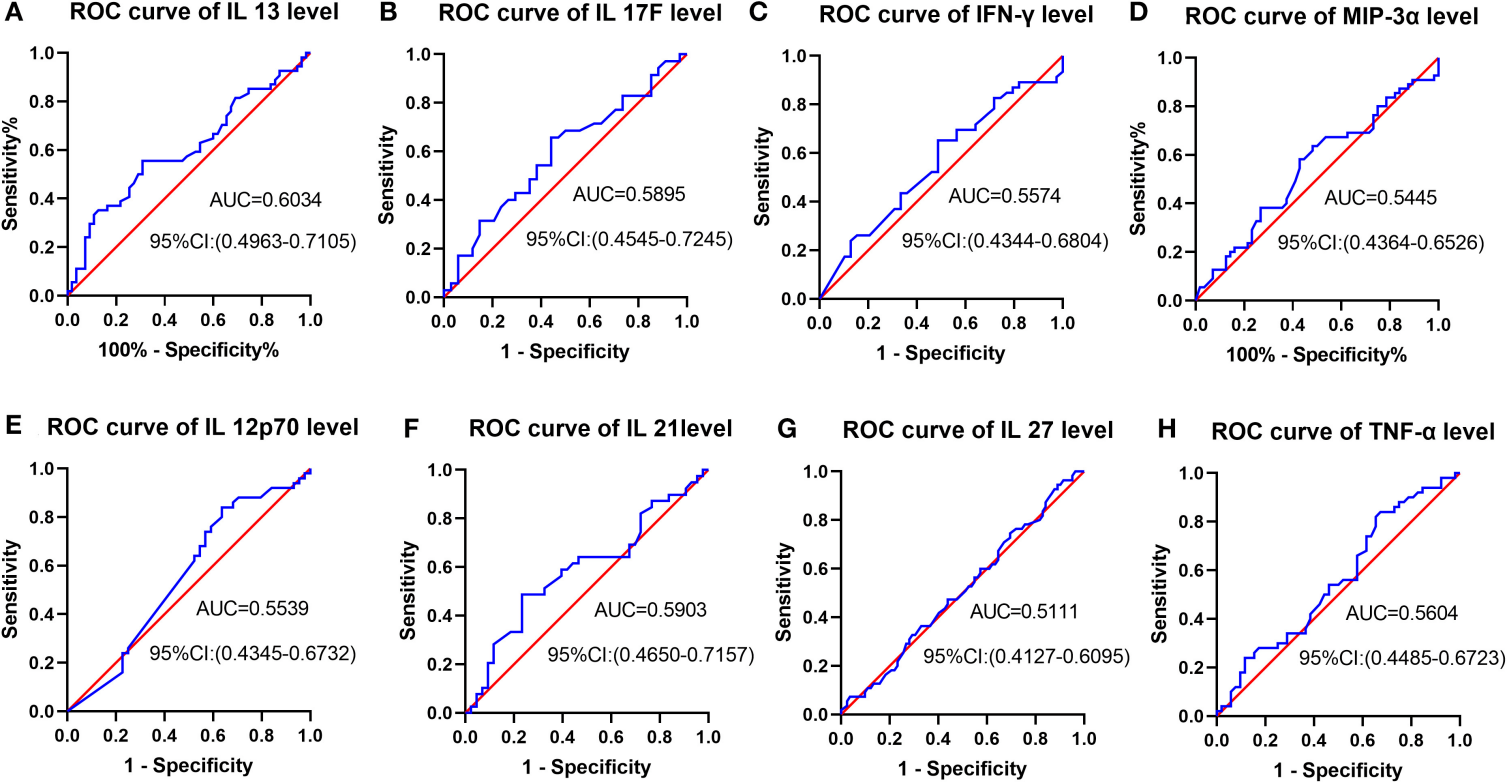
ROC analysis of cytokines as a predictor for post-stroke cognitive recovery improvement. **(A–H)** AUC is from 0.5111 to 0.6034, suggesting a low accuracy of prediction.

Figure 3 indicated receiver operating characteristic (ROC) analysis of cytokines as a predictor for post-stroke cognitive recovery improvement. From Figures 3A–H, the area under the curve (AUC) is from 0.5111 to 0.6034, suggesting a low accuracy of prediction.

### Post-stroke cognitive recovery and serum cytokines levels

**Figure 4:**
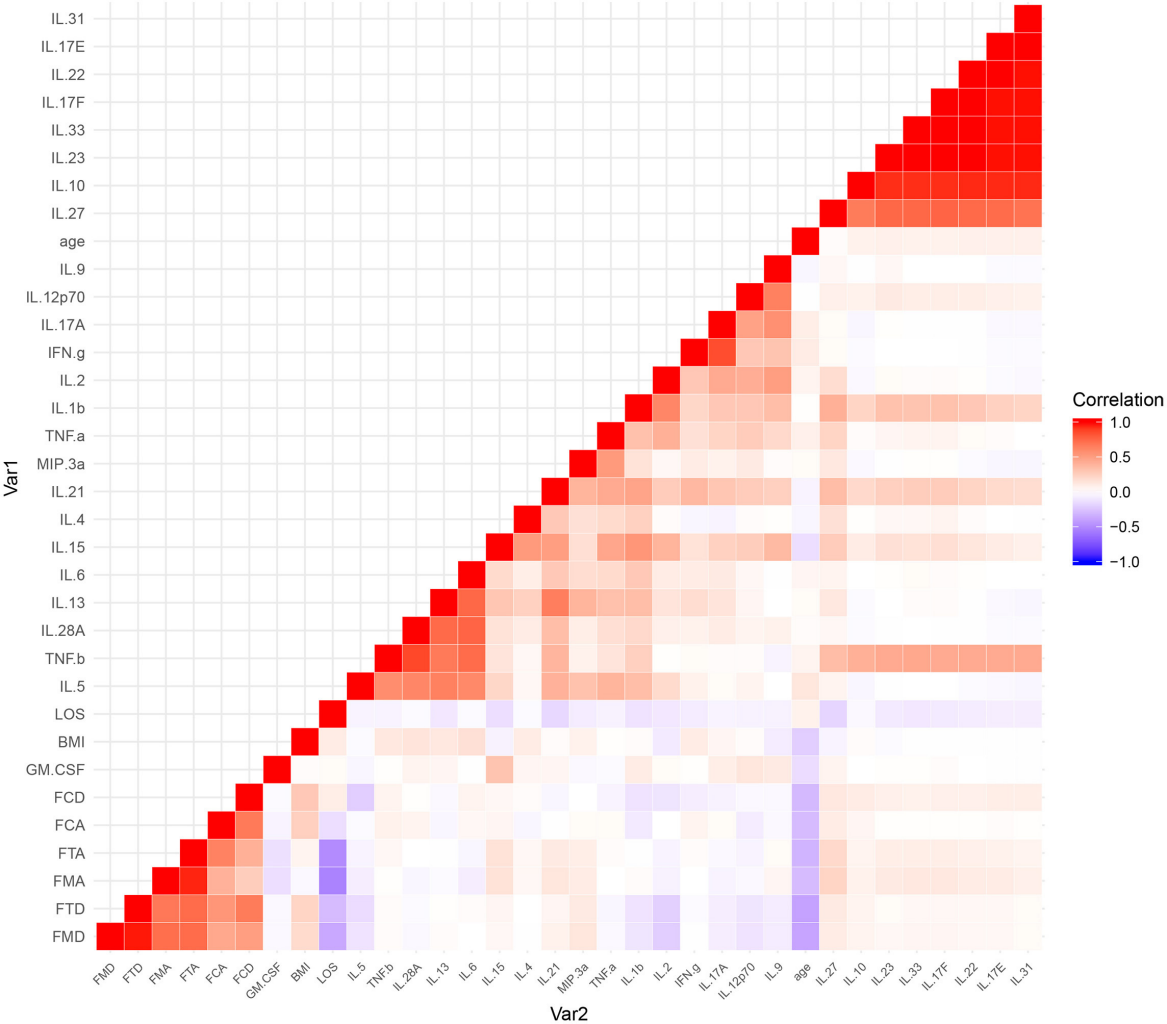
A heatmap of the correlational coefficients. Variables in graded shades of red (blue) represent their positive (negative) correlations. Representations of the correlation level are listed on the side of the graph. FCA, cognitive FIM score on admission; FCD, cognitive FIM scores at discharge; FMA, motor FIM score in admission; FMD, motor FIM scores at discharge; FTA, total FIM score on admission; FTD, total FIM scores at discharge; LOS, length of stay in hospital.

The results of the serum cytokine levels and clinical variables (age, BMI, gain of cognition, cognitive MRFS, cognitive MRFS efficiency, cognitive FIM scores on admission and at discharge, and length of stay in hospital) were represented in a graphical output, denoted as a heatmap (Figure 4).

Among a large set of variables, the heatmap utilizes color mapping to promote visual evaluation according to their correlation coefficients. Analytes in graded shades of red or blue represent their positive or negative correlations. In the heatmap, cytokines are grouped together based on their correlations, identified by visual indication as relatively monochromatic clusters.

However, no significant correlation was found between the cognitive variables (gain of cognition, cognitive MRFS, cognitive MRFS efficiency, cognitive FIM scores on admission and at discharge) and any of the serum cytokines (Figure 3). These results reflect that there is no significant correlation between cytokine levels and cognitive outcome.

The most pronounced results in the heatmap were two clusters of highly correlative cytokines (Figure 4). The larger cluster contained IL-10, IL-17E, IL-17F, IL-22, IL-23, IL-27, IL-31, and IL-33, and the smaller one contained IL-5, IL-6, IL-13, IL-28A, and TNF-β.

### Serum cytokines network

**Figure 5:**
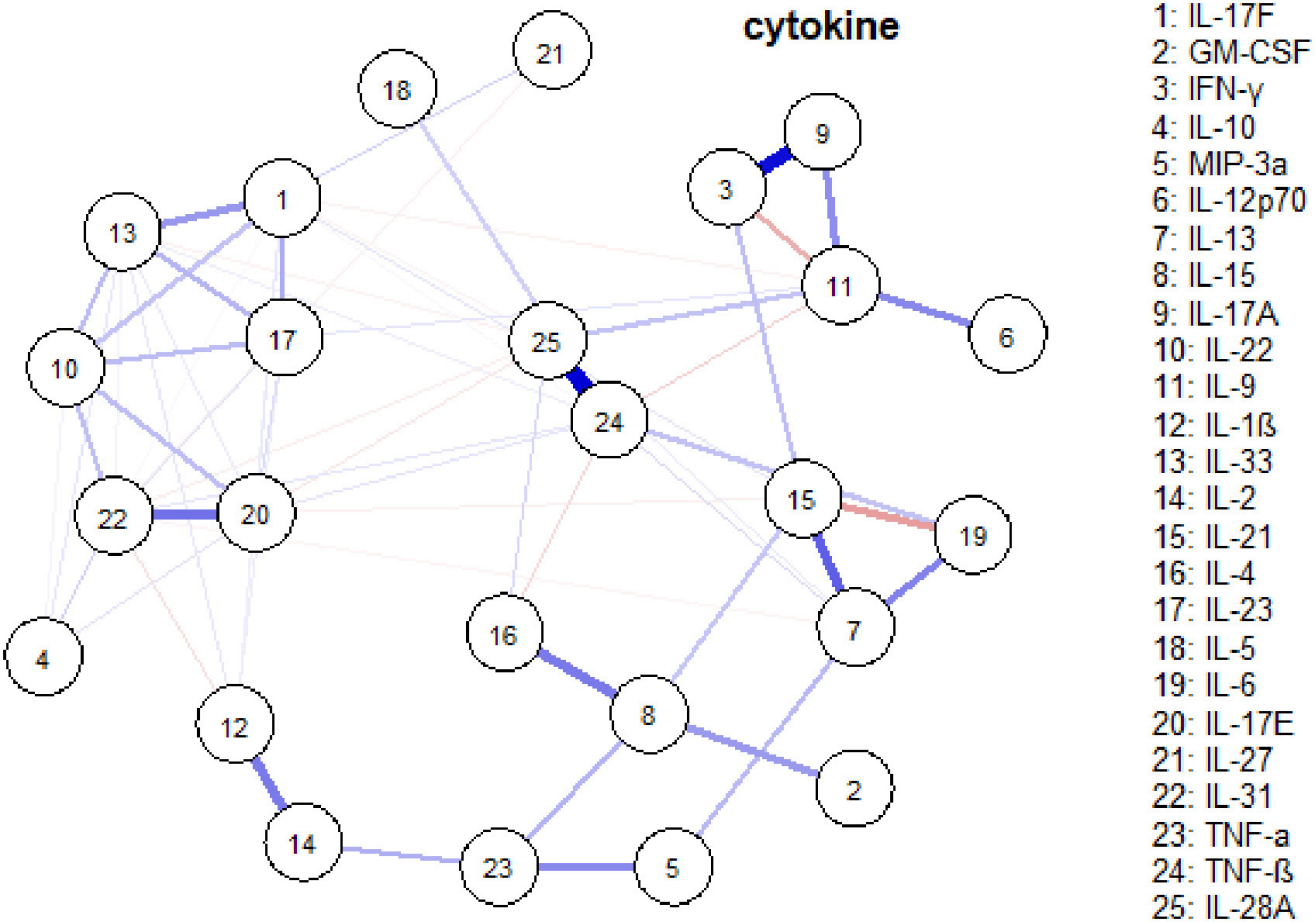
An estimated network structure of serum cytokines. The weight edges represent the strength of connections, while the blue (red) edges stand for positive (negative) relationships.

Network analysis was conducted to discover complex interrelationships of serum cytokines, which were represented in an estimated network structure. As shown in the estimated network structure of serum cytokines (Figure 5), some strong connections were found between TNF-β and IL-28A, as well as between IFN-γ and IL-17A. Similar correlations were found among IL-17E, IL-17F, IL-22, IL-23, IL-31, and IL-33, as well as among IL-6, IL-13, and IL-21. These are in concordance with the heatmap findings.

## Discussion

### Serum cytokines and post-stroke cognitive function

The objective of this study was to explore the association between serum cytokines levels and post-stroke cognitive outcome. The results suggest that serum cytokines alone offer minimal predictive value for cognitive function in the subacute phase after stroke.

Growing evidence suggests that inflammation is likely involved in cognitive impairment. In recent years, a number of studies have suggested that biomarkers, such as serum circulating cytokines in patients with PSCI could be a potential surrogate for the diagnosis and prediction of cognitive impairment (29). However, the relationship between cytokines and PSCI remains largely unclear.

Cytokines produced by macrophages in peripheral blood can pass through the blood brain-barrier (BBB) and therefore can further regulate brain neuroinflammation (30, 31). Cross-talks between peripheral inflammation and neuroinflammation have been reported (32). According to many experimental and clinical studies, the inflammatory process has an important role in the development of PSCI, inducing neuronal damage and loss of synapses that ultimately lead to cognitive impairment (33). Several studies have investigated the association between inflammatory markers and PSCI, with ambiguous results. For examples, some studies suggest that inflammatory cytokines such as IL-8, IL-12, and ESR were closely associated with PSCI. However, the relationship was not found between the IFN-gamma and TNF-α. There is no clear consensuses regarding the relationship of IL-1β, IL-6, IL-10, and CRP with PSCI either. The discrepancy may stem from several factors. Serum levels of cytokines can fluctuate due to many conditions (34), such as delayed blood processing and storage condition (35, 36). The nonspecific nature of the inflammatory cytokines may limit the effectiveness of reflecting the underline biological process of cognitive impairment (37). Results from this study are in-line with the school of thought that even though inflammation may be involved with PSCI, the level of individual serum cytokines may not be a strong predictor for PSCI, at least for the subacute phase of stroke.

### Cytokines interrelationship analysis helps to select target cytokines as biomarkers in PSCI

Although 25 serum cytokines have no significant relationship with the cognitive variables, the present study further demonstrated that the estimated cytokines network and heatmap help to reveal their complex interrelationships in PSCI.

Interestingly, we have identified two special subsets of cytokines that significantly correlated with each other. We have also pinpointed that IFN-γ and IL-17A, as well as TNF-β and IL-28A, have the strongest relationship. Therefore, according to the cytokine network and different cytokine profiles, selecting panels of cytokines as potential biomarkers will be worth investigating.

Among these cytokines, some have well-known interrelationships; whereas the correlation for others has not yet been well described. Th17 cells generate or involve in the development of Th17-related cytokines, such as IL-17 (IL-17A–IL-17F), IL-21, IL-22, and IL-23 (38). The IL-23/Th17 signaling pathway plays a critical role in the pathogenesis of some inflammatory diseases (39). IL-17A and IFN-γ could synergistically promote macrophage anti-infection immunity against stroke-associated pneumonia and acute pneumonic plague (40, 41). IL-17A and IFN-γ, as inflammatory mediators, could also disarray tight junctions (TJs) and rapidly improve the permeability of blood–brain barrier (BBB), implicating in the pathogenesis of CNS diseases (42).

After ischemia stroke, the IL-33/growth stimulation expressed gene 2 (ST2) pathway was activated, polarizing beneficial M2 macrophage and subsequently reducing neuronal cell death (43). IL-10 mediated the neuroprotective effects of the ST2/IL-33 signaling pathway (44). IL-33/ST2 pathway is also involved in Th2/IL-31 and Th17/IL17A immune responses in allergic airway disease (45). IL-33 expression increased after cell death and resulted in the induction of IL-31, implying a strong association between IL-31 and IL-33 in serum and tissue. The imbalance of IL-31 and IL-33 levels played a role in the inflammatory disorders (46). IL27 and IL31 from the IL-6 family could elicit essential reactions to modulate immune homeostasis, metabolism, hematopoiesis, inflammation, and development (47). IL-27 signaling could promote type 1 immune responses and directly limit IL-17 immunity, mitigating autoimmune diseases. However, IL-27 also interferes with IL-33 signaling, as a negative mediator of type 2 responses (48).

IL-5 plays beneficial roles in the repair of brain damage, suppresses post-stroke inflammation, and has a the capability to induce neurotrophic factors in astrocytes (49). IL-6 has dual function on ischemia stroke. It acts as an inflammatory regulator in the acute phase and as a neurotrophic regulator in the subacute and sequela phases (50). IL13 behaves as an anti-inflammatory cytokine, regulating microglia and macrophage post-stroke responses. IL13 is the well-characterized promoter of M2 polarization in microglia and macrophages, inducing a M1-to-M2 phenotype shift in the subacute phase of stroke (51). In the early stage of ischemic stroke, macrophages can acquire a protective function known as the anti-inflammatory (M2) phenotype, polarized by IL-13 or IL-4, and produce anti-inflammatory cytokines such as TGF-β (52) and IL-10 (53). Moreover, IL13 serves as an excellent candidate for more effective modulation of inflammatory responses in ischemic stroke, strongly decreasing the pro-inflammatory cytokine secretion, reducing inflammatory cell infiltration, and suppressing axonal loss (54). However, few studies have explored their synergetic relationship in regulating inflammatory balance after stroke. Their correlation gives a new insight into potential therapeutic applications. The relationship between IL-28A and TNF-β was not well clarified, which was worth further investigation.

GM-CSF has neuroprotective properties in stroke. GM-CSF cytokine level in this study showed a slight difference between the two groups. However, the detectable rate (3.45% in the poor cognitive recovery group and 5.36% in the good cognitive recovery group) was too low for further analysis. Cytokines with more than 50% detectable rate were compared and analyzed in this preliminary study. In future study, the GM-CSF level in peripheral serum may need to be measured using higher sensitivity method, such as a single molecule array (SIMOA).

As circulating cytokines probably induce broad immune inflammation processes in patients with post-stroke, a cytokines network may help to enhance the predictive value of cytokines as potential biomarkers. Correlations between serum cytokines concentrations indicate the co-regulation of immune inflammation.

In this preliminary study, some cytokines show positive correlations with other cytokines. In addition, some cytokines have high detectable rates, while others have quite low rates. In subset 1: the detectable rates of IL-10, IL-17E, IL-22, IL-23, and IL-31 are lower than 20% while the detectable rates of IL-17F and IL-27 are around 60 and 95%. The highest detectable rate in subset 2 is IL13 (around 95%). Therefore, we propose that the selective measurement of panels of cytokines might be cost-effective for serum cytokines studies.

### Limitations

The present study has several limitations. First, the cohort consists of patients with stroke from one urban rehabilitation hospital; therefore, the conclusion cannot be generalized to the general population. Second, the FIM cognitive subscore is the only cognitive measurement used to evaluate patients' cognitive impairment and is not very sensitive to subtle cognitive impairment.

Third, it is possible that the different blood sampling times could influence the cytokine levels and may have an impact on the study eventually. All the blood samples in this study were collected in the first week after admission to the rehabilitation hospital with variable interval to the onset of stroke. Future studies would need to add blood samples at the time of stroke onset to evaluate the time course of cytokine level and cognitive function.

Fourth, LUMINEX^®^multiplex array analysis has a limitation of sensitivity. Some cytokines were undetectable. In Ormstad's study, the detectable rates of cytokines using a Luminex system and multiplex array analysis varied from 4 to 93%, which was similar to our study (55). Multiplex is much less robust when using serum or plasma samples than tissue samples (56); multiplex assays, which theoretically can measure up to 100 analytes simultaneously, may result in potential interactions between different antibodies or cytokines in one assay. The high cost and possible low sensitivity also need to be considered in the application of multiplex arrays (57). As we mentioned in the previous section, selective measurement of cytokines would be a reasonable option to consider.

## Conclusion

Multiplex array analysis of serum cytokines offers minimal predictive value for cognitive function in the subacute phase after stroke.

## Data availability statement

The original contributions presented in the study are included in the article/Supplementary material, further inquiries can be directed to the corresponding authors.

## Ethics statement

The studies involving human participants were reviewed and approved by Spaulding Rehabilitation Hospital. Written informed consent from the patients or patients legal guardian/next of kin was not required to participate in this study in accordance with the national legislation and the institutional requirements.

## Author contributions

XL and QW were responsible for the study conception and design, resources, funding, overseeing data collection, data interpretation, critical review, and manuscript. YZ participated in the study design, performed data analyses, interpreted results, and contributed to manuscript processing and revision. HS and LW participated in its design and serum assays. XX participated in the analyses and interpretation of results. PC and JW contributed to data interpretation and critical review. All authors read and approved the final article.

## Funding

XL and JW are supported by the Shenzhen Science and Technology Innovation Program JCYJ20180305163652073.

## Conflict of interest

The authors declare that the research was conducted in the absence of any commercial or financial relationships that could be construed as a potential conflict of interest.

## Publisher's note

All claims expressed in this article are solely those of the authors and do not necessarily represent those of their affiliated organizations, or those of the publisher, the editors and the reviewers. Any product that may be evaluated in this article, or claim that may be made by its manufacturer, is not guaranteed or endorsed by the publisher.

## Abbreviations

PSCI: post-stroke cognitive impairment
CVA: ischemic cerebrovascular accident
FIM: functional independence measure
ROC: receiver operating characteristic
AUC: area under the curve
MRFS: Montebello Rehabilitation Factor Score
TNF-α: tumor necrosis factor alfa
IL: interleukin
GM-CSF: granulocyte macrophage-colony stimulating factor
IFN: interferon
MIP: macrophage inflammatory protein
Simoa: single molecule array.
