# Supplementary material for: Multiplex array analysis of serum cytokines offers minimal predictive value for cognitive function in the subacute phase after stroke
Source: Front Neurol. 2022 Oct 18;13:886018. doi: 10.3389/fneur.2022.886018 (PMC9622930; doi:10.3389/fneur.2022.886018)
Supplement: Supplementary file 1 [file Table_1.docx]

**Supplementary**

Table 1 The threshold for analyte detection

| Analyte(pg/ml) | IL-1 β | IL-2 | IL-4 | IL-5 | IL-6 |
| --- | --- | --- | --- | --- | --- |
| Threshold | 4.88 | 12.21 | 24.41 | 6.10 | 2.44 |
| Analyte(pg/ml) | IL-9 | IL-10 | IL-12p70 | IL-13 | IL-15 |
| Threshold | 8.54 | 1.22 | 4.88 | 7.32 | 4.88 |
| Analyte(pg/ml) | IL-17A | IL-17E | IL-17F | IL-21 | IL-22 |
| Threshold | 12.21 | 488.28 | 24.41 | 4.88 | 36.62 |
| Analyte | IL-23 | IL-27 | IL-28A | IL-31 | IL-33 |
| Threshold | 366.21 | 60.59 | 48.82 | 48.82 | 4.88 |
| Analyte(pg/ml) | GM-CSF | IFN-γ | MIP-3α | TNF-α | TNF-β |
| Threshold | 60.59 | 9.77 | 4.88 | 2.44 | 36.62 |
